# Mental imagery of whole-body motion along the sagittal-anteroposterior axis

**DOI:** 10.1038/s41598-022-18323-4

**Published:** 2022-08-23

**Authors:** K. Patel, D. Beaver, N. Gruber, G. Printezis, I. Giannopulu

**Affiliations:** 1 School of Human Sciences and Humanities, University of Houston, Houston, 77001 USA; 2 Faculty of Health Sciences and Medicine, Bond University, Gold Coast, 4226 Australia; 3 Department of Mathematics, University of Innsbruck, 6020 Innsbruck, Austria; 4 VASCage, 6020 Innsbruck, Austria; 5 Department of Electrical Engineering, Technological University of Denmark, 2800 Kgs. Lyngby, Denmark; 6 Creative Robotics Lab, UNSW, Sydney, 2021 Australia; 7 Clinical Research and Technological Innovation, 75016 Paris, France

**Keywords:** Psychology, Human behaviour, Neuroscience, Cognitive neuroscience, Perception

## Abstract

Whole-body motor imagery is conceptualised as a mental symbolisation directly and indirectly associated with neural oscillations similar to whole-body motor execution. Motor and somatosensory activity, including vestibular activity, is a typical corticocortical substrate of body motion. Yet, it is not clear how this neural substrate is organised when participants are instructed to imagine moving their body forward or backward along the sagittal-anteroposterior axis. It is the aim of the current study to identify the fingerprint of the neural substrate by recording the cortical activity of 39 participants via a 32 electroencephalography (EEG) device. The participants were instructed to imagine moving their body forward or backward from a first-person perspective. Principal Component Analysis (i.e. PCA) applied to the neural activity of whole-body motor imagery revealed neural interconnections mirroring between forward and backward conditions: beta pre-motor and motor oscillations in the left and right hemisphere overshadowed beta parietal oscillations in forward condition, and beta parietal oscillations in the left and right hemisphere overshadowed beta pre-motor and motor oscillations in backward condition. Although functional significance needs to be discerned, beta pre-motor, motor and somatosensory oscillations might represent specific settings within the corticocortical network and provide meaningful information regarding the neural dynamics of continuous whole-body motion. It was concluded that the evoked multimodal fronto-parietal neural activity would correspond to the neural activity that could be expected if the participants were physically enacting movement of the whole-body in sagittal-anteroposterior plane as they would in their everyday environment.

## Introduction

One of the most salient aspects of the human mind is the ability to mentally replicate actions without the need to physically execute them^1^. Building on from the “simulation hypothesis”, accumulating evidence suggests that motor imagery (MI), which encompasses a cognitive representation of an actual movement has similar neural correlates to those of real movement^2^. MI of distinct body parts simultaneously actuates and engages the equivalent sensorimotor and somatosensory cortices, including kinaesthetic information^3,4^. Empirical evidence for the existence of neural networks has focused particularly on signals associated with frontal (supplementary motor area, motor cortex, dorsolateral premotor cortex, inferior frontal gyrus), and parietal areas (primary somatosensory gyrus, superior and inferior parietal regions including vestibular cortex)^5,6^. Even though research supports the assumption that the representations underlying the imagery of whole-body motion share analogous mechanisms with the representations of real whole-body motion^7,8^, the dynamic oscillations of neural representations associated with the MI of whole-body remain less explored. The present study aimed to identify the fingerprint of these neural representations. To that end, the brain oscillations associated with the motor imagery of the body movement along the sagittal (i.e. anteroposterior) axis were recorded using electroencephalography (EEG).

Among all sensory contributions, vestibular contribution is predominant for allowing whole-body motion^9–15^. Under normogravity conditions, angular acceleration is transduced by the semicircular canals^16^, whereas linear acceleration, the position and orientation of the body in 3D space, are analysed and represented by the otoliths^15,17^. Neural similarities between the imagined and actual egocentric perspectives were greater during passive translation than during rotation (cited by van Elk and Blanke)^18^. In reference to the three body axes (sagittal, lateral and vertical) and during real^12^, virtual^19,20^ and imagined whole-body motion^21^, the vestibular system, together with the visual, sensorimotor and somesthetic systems, have also been reported to interact irrespective of the movement being global (involving the whole-body^14,22^) or segmental (involving specific body parts^23^). Evidence from theoretical and computational studies suggests that not only the peripheral (otoliths and semicircular canals) but also the central vestibular system (temporo-parieto-insular junction) internally simulate the whole-body motion^24–26^, and orientation in space, and integrate pathway-specific neural representations^27^. Such representations model the ongoing state of the visuo-vestibular, sensorimotor and somesthetic systems as well as the associated position and direction of the body to anticipate the next state^27^.

Previous studies have shown that when participants were instructed to adjust their body position and orientation on a vertical axis with respect to the earth’s horizontal and vertical axes, they behaved as if they were able to “*virtually perceive*” their internal model of gravity demonstrating that in real and imagined whole-body motion, participants compared their body perception based on vestibular feedback^11^. Highlighting the importance of vestibular inputs on whole-body motion, individual vestibular variability along the vertical axis was found to be associated with individual variability of whole-body motion along this axis^14^. The performance of healthy participants in microgravity^29^ and patients with vestibular dysfunctions was severely affected during real and imagined whole-body motion^30,31^. In addition, patients with bilateral vestibular ablation showed a significantly higher threshold than normal for real body motion around the vertical axis (i.e. yaw rotation), and along vertical and lateral translational axes^9^. Using chronometric tasks, some studies demonstrated that when healthy participants had to imagine walking to a previously perceived location, real and imagined walking durations were very similar^32^. On the contrary, some others revealed that imagined walking durations were quicker than real walking durations^33^. Whole-body motion chronometry along the sagittal axis was longer than along the vertical axis^34^, and did not differ between forward and backward sagittal translation^35^. On the other hand, linear distance reproductions in the dark after passive robotic translation were significantly misled^12^. Even if the heterogeneity in methodology can explain the source of the differences observed between real, virtual and imagined body motion, the aforementioned findings suggest that vestibular information continuously contributes to whole-body motion along the sagittal, vertical and lateral translational axes. Additionally, some evidence exists, which suggests that the production of real and imagined passive or active body motion necessitates the embodiment of motor, vestibular and proprioceptive/somesthetic information^36,37^. In this context, the motor imagery of the whole-body is to be analysed from a first-person perspective with priority given to premotor, sensorimotor, vestibular and kinaesthetic components. Body motor imagery is also to be considered as a substrate of real body motion. Consistent with this is the idea that MI induces somatosensory reduction in an equivalent way to real movement, and engages in essence the same forward models to predict the sensory consequences of imagined movements that occurs during overt movement^1^. On the corticocortical ground, MI of the whole-body appears to be organised around several sensorimotor brain regions: premotor, motor and primary post central gyrus, superior and inferior parietal cortices^38–40^.

The present study analysed possible modulations in the neural activation patterns engendered by mental imagery of whole body motion along the anteroposterior axis in healthy participants. The objective was to disentangle whether mental imagery of forward and backward body motion structures oscillatory neural signatures during the MI process. This study concentrated on specific regions of interest (ROIs), that is, corticocortical frontal (i.e. premotor, motor, inferior frontal gyrus), central (i.e. sensorimotor), parietal (i.e. primary somatosensory area, parietal superior and inferior gyri) organisation also associated with vestibular afferences that previous studies have reported to be imperative for whole-body simulation processes on the sagittal translational axis^41–43^. Based on previous studies demonstrating the top-down facilitating effect of mental preparation on whole-body sagittal motion^14^, first, healthy participants were involved in a mental preparation phase during which forward and backward (i.e. anteroposterior) whole-body translations were explained and performed by the experimenter using a mobile chair alone or when sitting in the chair. In order to ensure that the participants had understood the movements, they were asked to display the movement of the chair on the anteroposterior axis alone and whole-body motion while maintaining their seated position in the chair. Then, sitting in the chair and facing a computer screen, the participants were instructed and trained to imagine moving forward and backward from a first person perspective according to a defined motor imagery paradigm. Their brain activity was recorded on a 32 EEG electrode device. Considering that the forward whole-body motion is a quotidian anticipatory action which is highly embodied, embrained^9^ and grounded^44,45^ it is obvious that there is much to investigate in the brain’s interconnections within and between frontal, central and parietal areas (i.e. ROIs) during the mental imagery motion. Specifically, forward motor imagery, as an anticipatory movement, in essence, involving simultaneous visual feedback of self-imagined action, would increase anterior frontal neural activity that would attenuate posterior parietal activity; similarly backward motor imagery as it does not involve simultaneous visual feedback would increase posterior parietal activity that could in turn weaken anterior frontal activity.

## Material and methods

### Participants

The feasibility of the study was tested using G*Power 3.1 and the results have shown that the minimum number required was 34 participants to reach an adequate statistical power of 0.95 with a medium effect size (d = 0.50) and alpha level of 0.05. Forty volunteers, 20 males and 20 females were recruited. Due to technical issues, only 39 participants (19 males and 20 females) were retained for the final analysis. The mean age was 24.04 years (SD 3.84 years). The participants were undergraduate and postgraduate students from Universities in Australia. They were all from medium to high socio-economic backgrounds and voluntarily participated in the study. Each participant was rewarded with a AU$ 50 gift card. All participants declared they had a normal or corrected-to-normal vision and no history of vestibular, cardiac or neurological disorders. The study was approved by the University Human Research Ethics Committee (BUHREC 16065) and conformed to the National Statement and the Declaration of Helsinki. All subjects provided an informed consent prior to the experiment. Anonymity was guaranteed.

### Procedure

The procedure consisted of two baselines and three phases: mental preparation, training and experimental phase. The two baselines occurred before and after the training phase.

### Mental preparation phase

**Figure 1:**
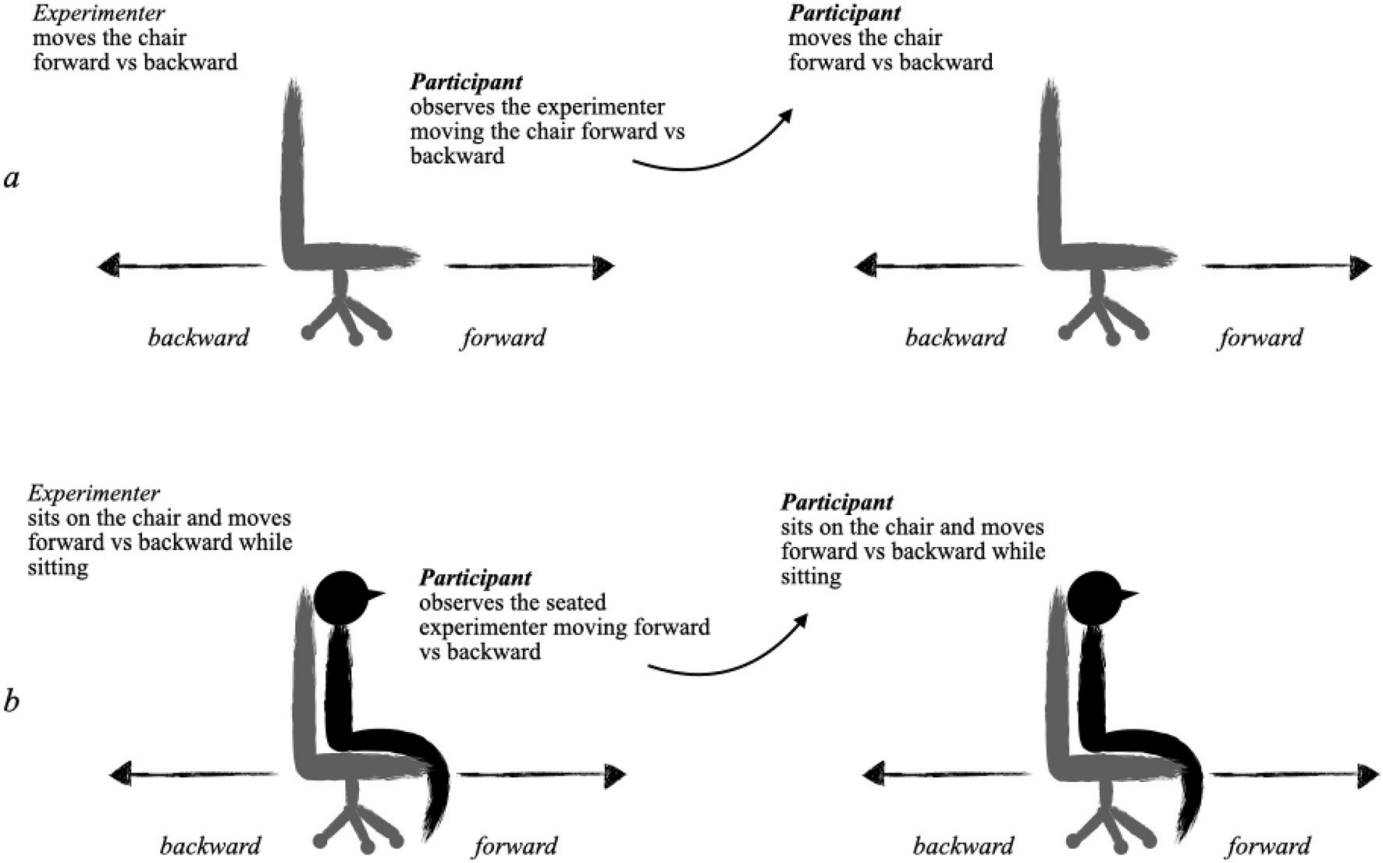
Schematic presentation of the mental preparation in two steps. (**a**) The experimenter moves the chair forward and backward in front of each participant first, and ask the participant to do the same immediately after; (**b**) the experimenter sits in the chair and moves forward and backward while sitting, and asked each participant to repeat the same.

The mental preparation phase aimed to facilitate participants’ understanding of the task to perform (Fig. 1). This phase began once participants declared themselves ready to start the study. First, each participant was asked to observe the experimenter moving a chair forwards and then backward. The experimenter then instructed each participant to move the chair themselves when standing. Once this preparation finished, the experimenter sat in the chair and demonstrated and named each of the movements. Finally, the experimenter asked each participant to sit directly in the chair and mimic the instructions. According to the defined criteria, only participants who performed this phase perfectly were included in the study.

### Two baselines: baseline before and after the training phase

During both baselines, each participant’s position was adjusted in front of the black screen, i.e. his/her line of sight was in the middle of the screen, the participant’s back and shoulders were in contact with the back of the chair^46^. Each participant was instructed not to move his/her head and body when facing the screen. The participant’s EEG was recorded in that position for 1 min in the dark. The first baseline occurred before the training phase, the second baseline after the training phase. The inter-phase delay (baseline-training-baseline) was about 1 min.

### Training phase

**Figure 2:**
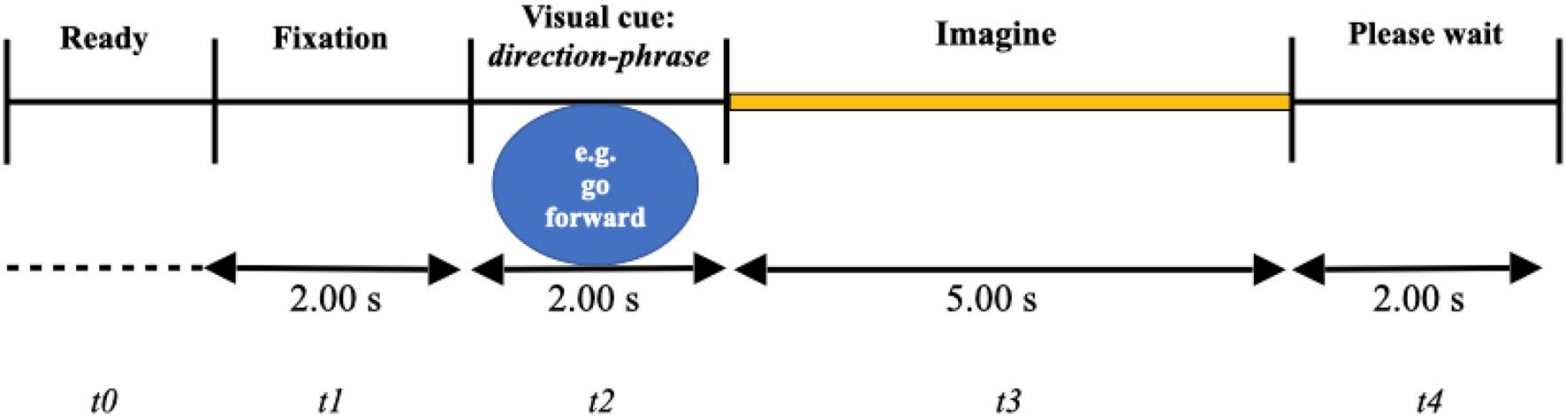
Timing presentation of the experimental paradigm of the whole body motor imagery. The start of each trial was signified by the presence of a short message “Ready”. This corresponds to *t* = *0.* After, a fixation point appeared in the middle of the screen for 2 s, i.e., *t* = *1,* fixation. After these 2 s, a direction-phrase indicating either "go forward" or "go backward" appeared for 2 s on the screen (i.e., *t* = 2). Once the words disappeared from the screen, each participant, facing the black screen, performed a motor imagery task, one at a time for 5 s, i.e., *t* = *3*. At the end of each motor imagery task, each participant was allowed to take a break "please wait", i.e. *t* = *4* for 2 s. In total, for 39 participants, there were 1,872 randomised trials. In 1,872 trials of 5 s each, resulted in 9360 s worth of data.

**Figure 3:**
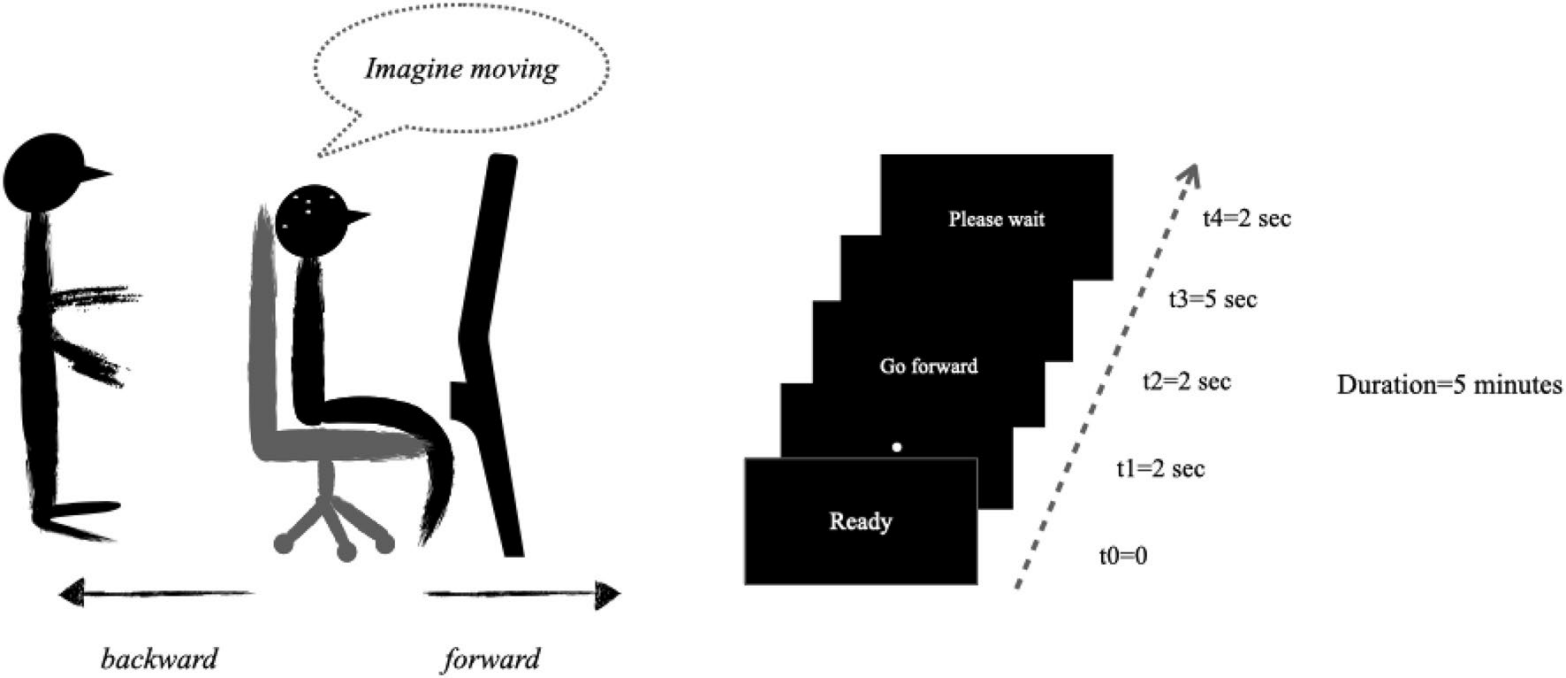
The experimenter instructed the participants to perform the mental imagery of whole-body motion from a first-person perspective: imagine move forward vs backward one at a time. To that end, the experimenter explained the paradigm to the participants step by step (i.e. from t = 0 to t = 4) verbally assisting each participant to respect the chronology and directives of each step (i.e. fix, read, imagine, relax) within the trials (i.e. experimenter-participant training). Each participant was also assisted to perform the task by him/herself as well (i.e. participant training). Participants were equipped with 32-electrode wireless EEG system.

The training phase aimed to familiarise each participant with the experimental paradigm. The experimental paradigm was a repetition of visual cue-based synchronous trials of different motor imagery tasks^4,47–49^. In accordance with this paradigm (Fig. 2), all participants were invited to imagine performing the whole-body motion task from a first-person perspective: move forward, backward one at a time. The experimenter explained the paradigm to the participants step by step (i.e. from t = 0 to t = 4) verbally encouraging each participant to respect the chronology and directives of each step (i.e. fix, read, imagine, relax) within the trials (i.e. experimenter-participant training). Each participant was also encouraged to perform the task by him/herself as well (i.e. participant training) (Fig. 3). Overall, the training phase lasted approximately 5 min with 3 min of experimenter-participant training and 2 min of participant training. At the end of this phase, each participant was asked to confirm whether s/he had clearly understood the task that they were to perform. Only participants who correctly performed the training phase and clearly confirmed understanding of the task were included in the study.

### Experimental phase

**Figure 4:**
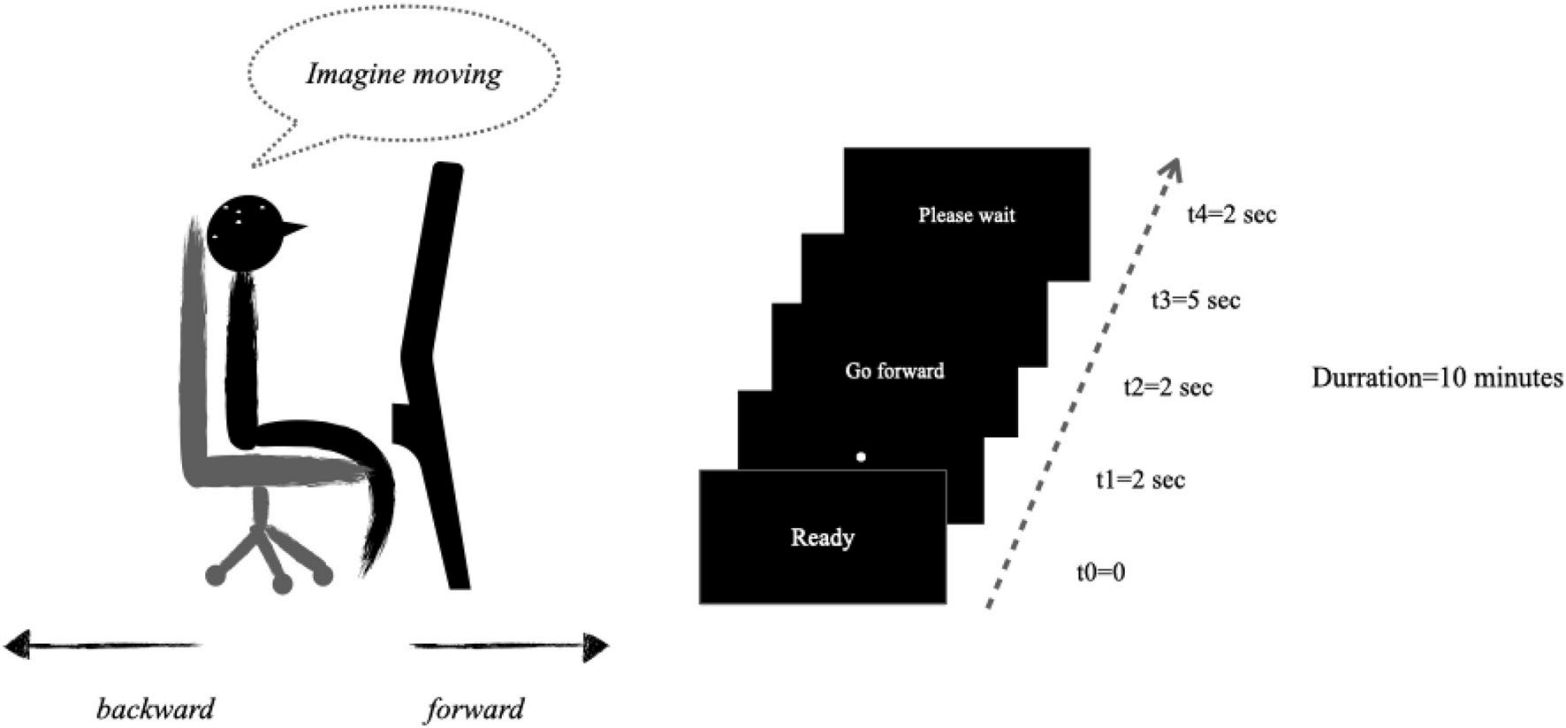
The participants were sitting in the same chair that s/he used for the mental preparation phase, the baseline and the training phase. Along with the training phase, each participant was adjusted in the front of an LCD screen monitor and instructed to imagine his/her own body motion when appropriate. The experimental paradigm was the same as presented in the training phase. All participants performed the mental imagery task in the dark. Participants were equipped with 32-electrode wireless EEG system.

As previously stated, during the experimental phase each participant was seated on the same chair that s/he used for the mental preparation phase, the baseline and the training phase. Along with the training phase, each participant was adjusted in the front of an LCD screen monitor and instructed to imagine his/her own body motion when appropriate (Fig. 4). The experimental paradigm was the same as presented in the training phase. A typical trial run was as follows: the start of each trial was signified by the presence of a short message “ready” in the middle of a black screen. This corresponded to *t* = *0.* Two seconds after, a fixation point appeared in the middle of the screen for 2 s, i.e. *t* = *1,* fixation point. Post the 2 s interval, a visual index, that is, a phrase, indicating either "go forward" or "go backward" appeared for 2 s on the screen. Each phrase (i.e. direction-phrase) constituted the visual cue (i.e.* t* = *2*) and, by definition, each corresponded to a whole-body movement direction. It required the participant to perform the corresponding motor imagery task, i.e. “go forward” or “go backward”. As such, once the words disappeared from the screen, each participant, facing the black screen, performed a motor imagery task, one at a time, for 5 s, i.e. *t* = *3,* imagery task (yellow colour). At the end of each motor imagery task, each participant was instructed to take a break and relax (i.e. please wait, *t* = *4*). The inter-trial interval was around 2 s. Each of the two types of motor imagery tasks was displayed 24 times within each sequence in a randomised order, i.e. 48 trials per participant. In total, for 39 participants, there were 1872 randomised trials. In 1872 trials of 5 s each, resulted in 9360 s worth of data, that is, 9,360,000 Hz of EEG data. The experimental phase was approximately 10 min for each participant.

### Data recording

EEG data recording was obtained using a 32 electrode EEG device (Mobita 32-electrode Wireless EEG System, Biopac^®^ Systems, Inc.) arranged according to the international 10/20 extended system: Fp1, Fpz, Fp2, F7, F3, Fz, F4, F8, FC5, FC1, FC2, FC6, T7, C3, Cz, C4, T8, CP5, CP1, CP2, CP6, P7, P3, Pz, P4, P8, PO, O1, Oz, and O2. The EEG was online band pass filtered between 0.1 and 100 Hz, and the data were digitised with a sampling rate of 1000 Hz. Mobita’s quality and reliability are given by Bateson et al.^50^.

### Data pre-processing and processing

Matlab R2021 (The Mathworks, Inc.) with FieldTrip toolbox was used to perform the data pre-processing and processing^51^. As per the experimental paradigm, only the 5 s of the mental imagery task were considered. The mental task was marked at the onset and the end of each condition (i.e. forward and backward motor imagery), each trial and each participant with a buffering of 500 ms before and after each 5 s period and a baseline correction − 400 to − 100 ms. A high-pass filter of 1 Hz and a low-pass of 80 Hz composed the pre-processing and processing script. Artefact detection was performed on all marked events. High-amplitude EEG artefacts, i.e. above 30 microvolts^52^, were automatically removed from all the events. All supplementary artefacts (i.e. electromyogram, electrooculogram and electrocardiogram activities) were eliminated manually after visual inspection by experts and corrected via independent component analysis (ICA) methods. Additionally, two independent experts visually controlled and manually eliminated all remaining artefacted events blind to the experimental condition. 95% constitutes the total percentage of retained trials, that is, 1.6% of EMG and 3.4% of EOG artefacted events were eliminated. The processing script performed a beta (13.5–30 Hz) frequency analysis on all filtered events. The 32 electrodes EEG device were organised into the following regions of interests (ROIs): frontal (i.e. F7, F3, Fz, F4, F8, FC5, FC1, FC2, FC6), central/fronto-parietal (i.e. C3, Cz and C4), and parietal (i.e. CP5, CP1, CP2, CP6, P7, P3, Pz, P4, P8 and PO) of the left and right hemisphere. The frequency analysis resulted in an average power spectrum measured in microvolts per Hertz (mV^2^/Hz) in each of the above mentioned ROIs for beta oscillations (13.5–30 Hz). For the statistical analysis, only the 5 s corresponded to the mental imagery task for forward whole-body motion on the one hand and backward whole-body motion on the other were considered (i.e. 5 s × 1872 trials for both condition).

### Statistical analysis

#### Beta oscillations comparisons between the ROIs areas

IBM SPSS Statistics 26 software was used to perform the statistical analysis. Prior to performing the analysis, several statistical assumptions (i.e. linearity, sphericity and normality assumptions) were verified. First, visual inspection of a scatterplot matrix demonstrated that linearity was met (i.e. data depicted a linear relationship). Similarly, sphericity analysis via Mauchly’s test taking into consideration a Greenhouse–Geisser epsilon (ε) showed non violation of this assumption. Normality assessment via the Shapiro–Wilk test showed all data distributions resulted in a level more than 0.05 signifying that the normality assumption was met (i.e. data followed a normal distribution). With that in mind, the comparisons between the averaged beta oscillations (13.5–30 Hz) of the ROIs areas (i.e. frontal, fronto-parietal and parietal) for forward on the one hand and backward motor imagery on the other was performed via parametric tests.

#### Principal component analysis (PCA)

To identify dynamic bilateral neural network patterns (i.e. correlograms) present during the mental imagery of forward and backward motion, the multivariate approach Principal Component Analysis (PCA) was performed as data suitability for a PCA was significant (Barlett’s test, p < 0.001)^53,54^. The average power spectrum associated with the beta oscillations for each trial, participant and experimental condition (i.e. forward vs backward) constituted the initial variables (i.e. inputs)^55^. As a dimensional reduction method, PCA creates relevant new variables (i.e. principal components) through linear combinations of the initial variables. The projection of these variables into the reduced PCA space is based on the generated eigenvectors of the correlation matrix (represented by the correlograms), each correlation is expressed with a coefficients of correlation called loading and is reported in each principal component. By definition, the loadings correspond to the eigenvectors multiplied by the square root of the corresponding eigenvalues. Based on the above, the performed PCA revealed various correlograms with each correlogram reflecting the temporal correlation of multiunit recordings associated with separable neural oscillations registered synchronously from the brain areas band of interest (13.5–30 Hz) and experimental condition (i.e. forward vs backward). The computations associated with the functional connectivity of the distributed brain activity was based on the identified correlograms.

## Results

### Forward vs backward comparisons

**Figure 5:**
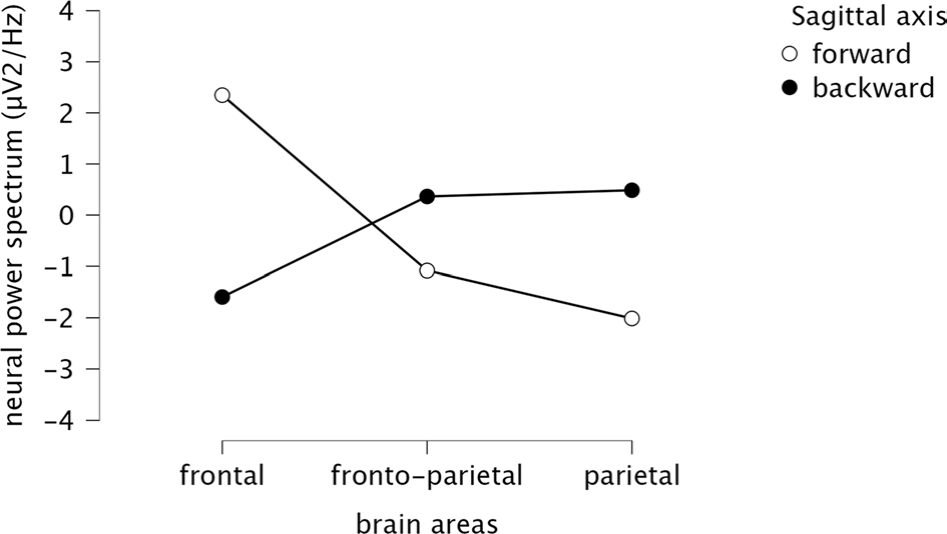
Comparison between forward and backward motor imagery (i.e. sagittal axis) in relationship with the average power spectrum in the three beta-frequency ROIs. X axis represents the selected ROIs (i.e. frontal, fronto-parietal and parietal); Y axis illustrates the average neural power spectrum (μV^2^/Hz) in beta-frequency (13.5–30 Hz), i.e. power spectrum of the neural activity.

The average power spectrum of the neural oscillations of anterior (frontal), central (fronto-parietal) and posterior (parietal) areas of beta-frequency was compared between forward and backward motion imagery. To reduce the risk of type I and type II errors^53^, the significant level for the ANOVA analysis was fixed at a = 0.10. Repeated measure ANOVA revealed a statistically significant effect of sagittal motion (forward vs backward) imagery on brain areas (frontal, fronto-parietal and parietal), F(2, 74) = 453, p < 0.001. As illustrated in Fig. 5, post-hoc comparisons reported a significant increase in power spectrum in frontal areas for forward (M = 2.35, SD = 0.87) compared to backward (M = − 1.59, SD = 0.74) motor imagery (t(38) = − 26.27, p < 0.001). When fronto-parietal areas were concerned, the power spectrum decreased for forward motor imagery (M = − 1.08, SD = 0.66), and increased for backward motor imagery (M = 0.36, SD = 0.08). This difference was statistically significant (t(38) = 9.64, p < 0.001). Similarly, we observed a statistically significant decrease in power spectrum in parietal areas for forward motor (M = − 2.01, SD = 0.99) compared to backward motor imagery (M = 0.49, SD = 0.12) (t(38) = 16.66, p < 0.001). Overall, the power spectrum of beta-frequency of the forward motor imagery mirrors the power spectrum of beta-frequency of the backward mental imagery and was higher for the frontal than for the fronto-parietal and parietal areas.

**Figure 6:**
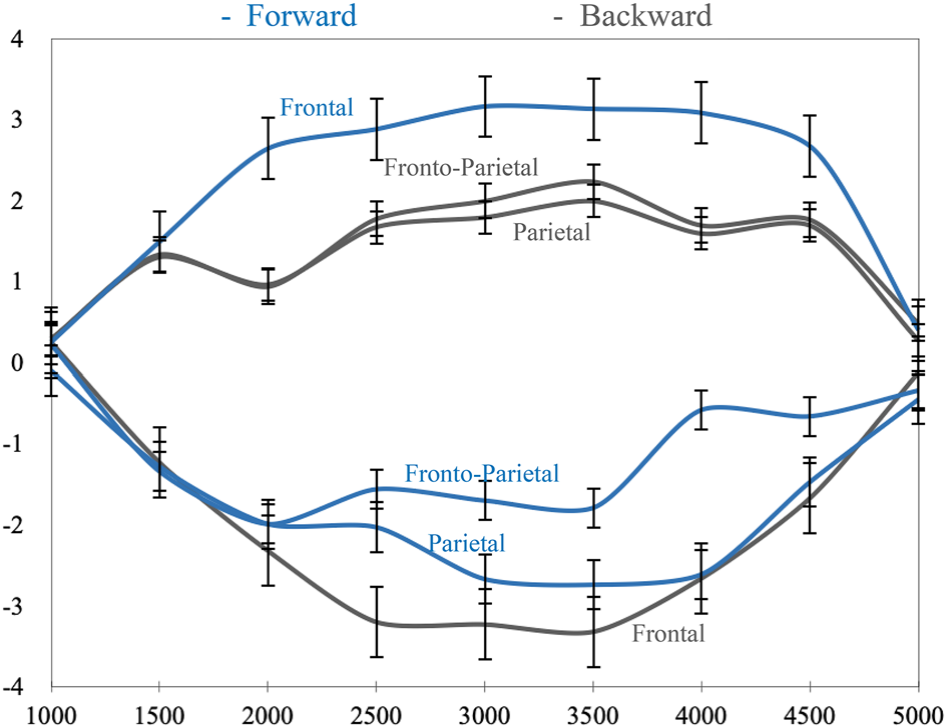
Temporal evolution of beta-frequency (13.5–30 Hz) over 5 s time period across the three ROI’s for forward and backward mental imagery (i.e. sagittal axis). X axis represents the time-course of 5 s in milliseconds based on a time-window of 1000 ms; Y axis illustrates the average power spectrum of the neural activity (μV^2^/Hz). Blue colour depicts "forward motor imagery"; grey colour illustrates "backward motor imagery".

The Fig. 6 represents the temporal evolution of beta oscillations over the 5 s across the three ROI’s for forward and backward motor imagery. Five time-windows of 1000 ms corresponded to the total duration time of the mental imagery task (5 s) were considered: 1000–2000 ms, 2001–3000 ms, 3001–4000 and 4001–5000 ms. The average power spectrum of the neural oscillations in anterior (frontal), central (fronto-parietal) and posterior (parietal) areas beta-frequency was calculated for forward and backward motor imagery. As previously, the significant level for the ANOVA analysis was fixed at a = 0.10, to minimise the risk of type I and type II errors^53^. Parametrical tests and more particularly repeated measure ANOVA reported a statistically significant effect of sagittal motor imagery (i.e. forward vs backward) on brain areas (frontal, fronto-parietal and parietal) for the defined time-windows (F(2, 87) = 175, p < 0.001). When post-hoc comparisons were considered, we observed a higher neural power spectrum in frontal areas for forward (M = 1.46, SD = 0.63) than for backward (M = − 1.10, SD = 0.45) motor imagery from 1000 to 2000 ms time-window (t(38) = − 36.43, p < 0.001) and, from 2001 to 3000 ms (M = 2.89, SD = 0.72 for forward and M =—2.05, SD = 0.43 for backward motor imagery, t(38) = − 23.51, p < 0.001). Similarly, the neural power spectrum was higher in frontal areas for forward (M = 3,12, SD = 0.72) than for backward (M = − 2.05, SD = 0.43) motor imagery from 3001 to 4000 ms (t(38) = − 15.76, p < 0.001) and from 4001 to 5000 ms (M = 2.05, SD = 0.62 for forward vs M = − 1.43, SD = 0.39 for backward motor imagery, t(38) = − 11.21, p < 0.001). The neural power spectrum decreased in fronto-parietal areas for forward motor imagery from 1000 to 2000 ms (M = − 1.04, SD = 0.29), from 2001 to 3000 ms (M = − 1.75, SD = 0.18), from 3001 to 4000 ms (M = − 1.36, SD = 0.43), and from 4001 to 5000 ms (M = − 0,53, SD = 0.46) compared to backward motor imagery (M = 0.85, SD = 0.21 for the first time-window, M = 1.56, SD = 0.13 for the second time-window, M = 1.97, SD = 0.66 for the third time-window, and M = 1.31, SD = 0.21 for the fourth time-window). Such observation was statistically significant (t(38) = 9.23, p < 0.001 from 1000 to 2000 ms, t(38) = 10.34, p < 0.001 from 2001 to 3000 ms, t(38) = 11.32, p < 0.001 from 3001 to 4000 ms, and t(38) = 19.64, p < 0.001 from 4001 to 5000 ms). The neural power spectrum in parietal areas was significantly lower for forward (M = -2.05, SD = 0.69) than for backward motor imagery (M = 0.85, SD = 0.69) from 1000 to 2000 ms (t(38) = 13.11, p < 0.001), from 2001 to 3000 ms (M = − 2.23, SD = 0.43 for forward vs M = 1.49, SD = 0.62 for backward motor imagery, t(38) = 19.16, p < 0.001); from 3001 to 4000 ms (M = − 2.67, SD = 0.47 for forward vs M = 1.79, SD = 0.69 for backward, t(38) = 26.51, p < 0.001); and from 4001 to 5000 ms (M = − 1.51, SD = 0.53 for forward vs for M = 1.18, SD = 0.61, backward motor imagery, t(38) = 33.72, p < 0.001). Overall, the neural power spectrum of the forward motor imagery and backward motor imagery reported systematically inverted effects from 1000 to 5000 ms.

### Functional connectivity: principal component analysis (PCA)

The predominantly dynamic patterns of beta oscillations brain connectivity during the mental imagery along the sagittal axis (i.e. forward vs backward) was explored via the multivariate approach PCA^54,55^. For the forward and backward experimental condition, the PCA analysis revealed three principal components reported eigenvalues greater than one. To facilitate the interpretability of these components, a Varimax rotation was performed as the correlation between components produced by the Factor Correlation Matrix (FCM) was smaller than 0.30^55^. Using a cut-off of 80% for inclusion of initial variables in interpretation of these three components, all initial variables loaded in those two components. These latter components captured at least 80% of the total sum of eigenvalues and corresponded to frontal and parietal brain areas.

**Figure 7:**
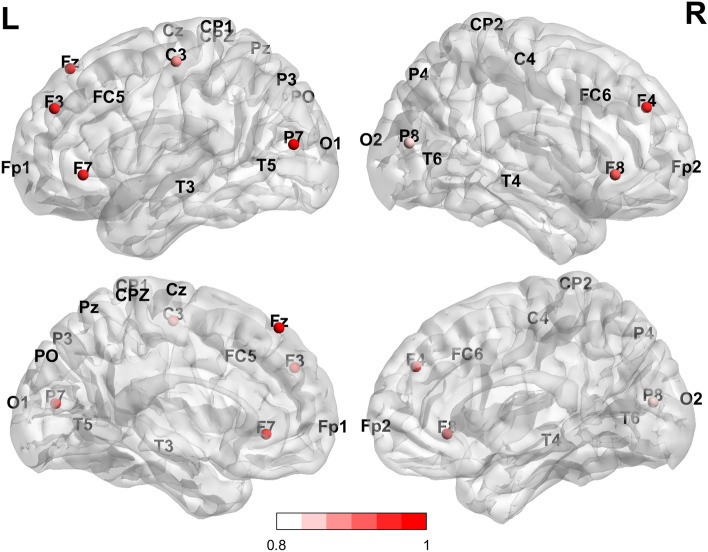
Loadings derives from PCA (Principal Component Analysis) for forward mental imagery. The PCA revealed two components accounting for 76% of the total variance. There was more beta synchronisation in frontal bilateral areas and less beta synchronisation in fronto-parietal unilateral left areas. Bilateral parietal synchronisation was also observed (Top: Sagittal external section; Bottom: internal sections. L: left hemisphere; R: right hemisphere).

For the forward motor imagery, the first principal component accounted for 76% of the total variance (Fig. 7). It was strongly correlated with six of the anterior electrodes (i.e. Fz, F4, F3, F7, F4 and F8) and one of the central electrodes (i.e. C3), that is, these electrodes vary together. More particularly, frontal connectivity patterns with large positive loading characterised the anterior part of the rostral body of the corpus callosum (i.e. Fz = 0.930), the premotor areas of the right (i.e. F4 = 0.924, F8 = 0.896) and left (i.e. F3 = 0.923, F7 = 0.923) hemispheres. Large positive loadings were also observed for the sensori-motor (i.e. C3 = 0.882) areas of the left hemisphere. Given the importance of the involvement of the anterior areas premotor areas of the left and right hemispheres, this first component was "premotor-frontal". The second component includes three posterior electrodes (i.e. P7, P8 and PO) with large positive loadings patterns connectivity of the parieto-temporal left (i.e. P7 = 0.934) and right (i.e. P8 = 0.841) hemisphere and also involves parieto-occipital neurotopology (i.e. PO = 0.806). Involving the posterior areas, this second component was "parietal". Overall, forward motor imagery predominately engages the anterior premotor areas (i.e. pre-motor areas) and also engages the parieto-temporal and parieto-occipital brain areas bilaterally.

**Figure 8:**
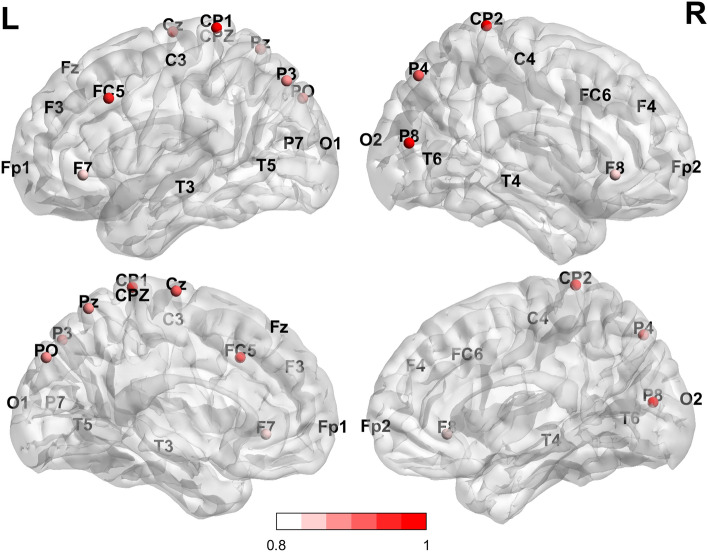
Loadings derives from PCA (Principal Component Analysis) for backward mental imagery. Two components which explained 94% of the total variance were reported. There was more beta synchronisation in parietal and fronto-parietal areas bilaterally and less beta synchronisation in frontal areas bilaterally and unilaterally left areas (Top: Sagittal external section; Bottom: internal sections. L: left hemisphere; R: right hemisphere).

The PCA for backward motor imagery revealed two components (Fig. 8). The first component accounted for 94% of the total variance and was strongly correlated with seven electrodes of the posterior brain areas (i.e. CP1, P8, CP2, P4, Pz, P3, and PO) and one electrode of the central brain areas (i.e. Cz). Specifically, correlated neural activity for backward motor imagery exhibited large positive loadings at the superior parietal somatosensory area (i.e. CP1 = 0.957) of the left hemisphere, inferior temporo-parietal areas of the right (i.e. P8 = 0.950) hemisphere, superior parietal somatosensory areas of the right (i.e. CP2 = 0.937) hemisphere, the middle of the body of the corpus callosum (i.e. Cz = 0.903) but also the superior parietal right (i.e. P4 = 0.896) hemisphere, the posterior part of the mid-body of the corpus callosum (i.e. Pz = 0.892), the superior parietal left (i.e. P3 = 8.77) hemisphere, and the bilateral parieto-occipital area (i.e. PO = 0.877). Clearly involving the parietal posterior brain areas, this component is "parieto-temporo-occipital". The second principal component includes three electrodes (i.e. FC5, F7 and F8) and involved large positive loadings for the motor area of the left (i.e. FC5 = 0.924) hemisphere and premotor frontal areas of the right (i.e. F8 = 0.823) and the left (i.e. F7 = 0.841) hemisphere. Based on its neurotopology, this component was "premotor frontal". Specifically, backward motor imagery fundamentally involves bilateral posterior somatosensory areas and also includes bilateral parieto-temporo-occipital areas.

## Discussion

The present study aimed to analyse the neural signature within the corticocortical fronto-parietal areas for forward and backward (i.e. anteroposterior) imagined whole-body motion according to a defined experimental paradigm. The participants were physically prepared for the motor imagery task by executing the movements along the anteroposterior axis (i.e. forward and backward) when seated in a chair. They were also mentally prepared to perform the task, before accomplishing the motor imagery task, that is, to imagine moving their body forward or backward from a first-person perspective for five seconds after the presentation of a direction-phrase (i.e. visual cue) displayed on the computer screen. The electrical neural activity of all participants was recorded via a 32 electrode EEG device placed in accordance with the international 10–20 system. Electromyographic (EMG) activity was not recorded during the forward and backward motor imagery due to recent studies demonstrating that the motor commands for muscle contractions during mental imagery tasks are blocked by the motor system^56,57^. However, the data was visually checked by experts and all artefacted events (i.e. EMG and EOG artefacts) were eliminated. Similarly, as reported in the pre-processing and processing session, ICA method was utilised in addition. Based on the assumption that each voluntary task potentially generates a specific brain activity that is linked to a variety of cognitive processes^58^, and considering that the forward whole-body motion as a quotidian anticipatory action is highly embodied and embrained, the mental imagery of forward motion, as a self-produced movement, was expected to engage more corticocortical anterior frontal neural activity than posterior parietal activity. Likewise mental imagery of the backward movement was expected to engage more corticocortical posterior parietal activity than anterior frontal activity as this movement is an unusual and unconventional action. Involving the movement of the whole-body in a mentally imagined process, signifies the implication of complex cortical connections. Accordingly, functional connectivity approaches were used to analyse beta oscillations corticocortical activity after having averaged and compared the power spectrum of beta oscillations between the defined ROIs and experimental conditions. More particularly, first parametric tests were utilised for the comparison of beta oscillations of the frontal, fronto-parietal and parietal areas between forward and backward motion imagery, and then the Principal Component Analysis (PCA) was conducted to identify the most important neural network patterns of beta oscillations in each experimental condition (i.e. forward vs backward). In essence, beta oscillations comparisons and functional connectivity measures (i.e. PCA) highlighted analogous modifications of neurocognitive processes, that is, imagining moving the whole-body forward or backward but supported complementary insights into neurocognitive processing.

When all comparisons were considered, the results revealed that the beta oscillations in the frontal bilateral areas increased in synchronisation for forward and decreased in synchronisation for backward whole-body motor imagery. They have also shown that the beta oscillations in the fronto-parietal and parietal bilateral areas decreased in synchronisation for forward whole-body motor imagery compared to backward whole-body motor imagery. Similarly, when the dynamic changes of the beta neural activity over the whole duration of the imagery task (5 s) across the three-ROI’s for forward and backward motor imagery was concerned, the results reported frontal beta suppression for backward motor imagery, and the reverse effect for forward motor imagery. They also reported fronto-parietal and parietal suppression for forward motor imagery in contrast to the backward motor imagery. These latter findings indicated systematic and significant dynamic changes from 1000 to 5000 ms and provided complementary insights into neurocognitive processes. Consistent with the hypothesis of the study, these findings indicate that the motor imagery of the whole-body along the anteroposterior axis (i.e. forward vs backward) mainly involves frontal (i.e. pre-motor and motor) activity that was more pronounced for forward than backward imagined motion, and fronto-parietal (i.e. sensorimotor) and parietal (i.e. somatosensory) activity that was more pronounced for backward than forward imagined motion. Expressly, the neurodynamic beta forward motor imagery oscillations mirrored the neurodynamic activity of beta oscillations associated with the backward motor imagery.

Expressing on a rostrocaudal continuum starting from the pre-motor and extending to the somatosensory neural activity, the results are coherent with existing data demonstrating that mental movement of the body activates neural correlates associated with frontal^59^ and parietal regions^16^ that are also reported to be interconnected with the vestibular afferential processing^23,27^. Frontal (i.e. pre-motor and motor) activity was significantly noticeable for forward than backward motor imagery. These results are consistent with recent findings according to which the pre-motor and motor areas are involved in action and action-control and orientation of the mental imagery task^60^. When the anteroposterior axis as a whole (forward and backward) was concerned, clear beta fronto-parietal oscillations (i.e. synchronised or desynchronised) were revealed during the required imagery task that is also consistent with data revealing that the diploid activation (i.e. frontal and parietal) during motor imagery of the whole-body authorises the renewal of spatial references^61^. Adapting to the current paradigm, the renewal of spatial references for forward motion was importantly associated with the beta anterior frontal oscillations and for backward motion was notably connected with beta posterior parietal oscillations. The differences between beta oscillations correlates for forward and backward whole-body imagery might mirror fluctuations in the motor action representations of the participants. Interestingly, recent magnetoencephalography (MEG) studies together with the application of artificial neural networks identified significant features of mental imagery in untrained participants^62,63^. Expressly, mental imagery of body parts (i.e. left and right arms) was classified into two types: visual imagery (VI) and kinaesthetic imagery (KI), where VI was associated with the visual component of the motor action, and KI was reported to be linked with the imagined muscle sensations of the motor action. The analysis of brain dynamics revealed that VI synchronisation neurotopography was aligned with the fronto-parieto-occipital activity, whereas the KI desynchronisation neutopography was associated with the infero-parietal activity. Given the neurotopographic similarities between the aforementioned studies and the current study, one can suggest that forward motor imagery with the inherent simultaneous visual feedback of self-imagined action principally involving frontal areas. This would be a typical manifestation of visual imagery. Subsequently, backward mental imagery fundamentally requiring parietal somatosensory areas would be a typical manifestation of kinaesthetic imagery.

When the patterns of dynamic neural network (PCA analysis) were considered, the results revealed that beta oscillations for forward whole-body motor imagery clearly involved bilateral frontal pre-motor areas (i.e. Fz, F4, F3, F7 and F8) that were highly interconnected and synchronised. Left unilateral beta oscillations of sensorimotor (i.e. C3) and bilateral somatosensory parietal cortex especially included parieto-temporal areas (i.e. P7 and P8) were also reported to be synchronised during the forward motor imagery of the whole-body. According to Engel and Fries^64^ changes in beta oscillations are mostly associated with top-down voluntary control to maintain a specific cognitive state. An increase in the synchronisation of the beta oscillations during the forward motor imagery might be associated with an integration mechanism between premotor, sensory and somatosensory (including vestibular) processes. This is coherent with studies, which reported that synchronisation in beta oscillations linked to conscious motor processing (real or imagined) engages neural cortical premotor^65^, sensorimotor^66,67^ and somatosensory activity^68^. Similarities between real motor execution and motor imagery oscillations at pre-motor, sensorimotor and parieto-temporal beta levels suggest that such neural activity is efferent and linked to the organisation of the ongoing movement^69^. Specifically, the beta synchronisation of the bilateral pre-motor areas (i.e. Fz, F4, F3, F8, F7) and left post central gyrus (i.e. C3) might correspond to the mental simulation of the volitional imagery of the forward whole-body motion with passive proprioceptive processing, whereas beta synchronisation of bilateral parieto-temporal areas (i.e. P7 and P8) might be considered as a "propagation effect" of the ongoing whole-body motor simulation that also includes passive vestibular afferences. Note that with the exception of the unilateral left primary sensorimotor area (i.e. C3), both pre-motor and parieto-temporal areas showed a clear bilateral, and thus, symmetrical beta oscillations involvement. Such bilateral beta oscillations should exhibit a somatotopic organisation of the imagined whole-body motion appearing to reflect analogous brain neural states in frontal and inferior parietal cortices regardless of whether a movement is prepared or mentally simulated^70^. Interestingly, frontal neural correlates (i.e. pre-motor) showed most important neurodynamic data dimension compared to the inferior parietal dimension (i.e. somatosensory). Consistent with the hypothesis of the current study and existing published research^71^, these findings suggest that mental imagery of forward whole-body motion, as a self-produced anticipatory action, might generate substantial pre-motor neurodynamic interconnections that would overshadow forward somatosensory neurodynamic interconnections. It appears from the above that beta oscillations correlates for forward whole-body imagery should necessitate a noticeable neural activation including the simultaneous visual feedback, to recreate the learnt body experience: commonly, constantly moving forward. This is consistent with recent data reporting that forward body motion tends to be more "grounded"^44,45^ as it is more highly embodied and embrained than backward motion.

Beta oscillations for backward motion imagery involved strong neural bilateral interconnections between superior parietal areas (i.e. CP1 and CP2) that are associated with adjacent bilateral parietal areas (i.e. P3 and P4) and also involve bilateral posterior parietal gyri (Pz), central sensorimotor gyri (i.e. Cz), parieto-occipital (i.e. PO), and right parieto-temporal area (i.e. P8). Beta neural interconnections were also secondary observed in the unilateral left motor area (i.e. FC5) and in bilateral inferior pre-motor areas (i.e. F7 and F8). Predominately involved somatosensory areas that are strongly associated with whole-body representations and accessorily revealed pre-motor areas, these results are consistent with the hypothesis of the study according to which backward mental imagery of the whole-body motion would increase the synchronisation of the beta parietal oscillations that, in turn, would attenuate the synchronisation of the beta frontal oscillations. In other words, beta oscillations associated with backward motor imagery can be recorded not only macroscopically, that is, motor and somatosensory, but also microscopically, that is, in pre-motor areas, which in the current situation were bilateral somatosensory and pre-motor and obviously linked with the parietal and frontal areas.

These results are coherent with several findings according to which the increase in amplitude observed in beta oscillations would be associated not only with the active movement control generated by an efferent input (i.e. motor) but also with the afferent proprioceptive feedback to the motor cortex during active and passive movement^72^. Especially, posterior somatosensory beta oscillatory interconnections overshadowed anterior motor and pre-motor beta oscillatory interconnections. Additionally, parieto-occipital (i.e. PO) areas were activated during the mental backward motion that were also reported to be induced by a specific sensory input modality^73^, which in the present case, was visual (i.e. direction-phrase presented on the screen). Specifically, the participants were instructed to imagine moving their body along the sagittal axis within the direction indicated by the visual cue depending on the condition (i.e. go forward or go backward). The observed cortical oscillations likely provide a temporal encoding of the represented somatosensory movements that undoubtedly contribute to the conscious motor action of the whole-body: moving backward. The current findings provide evidence of the fact that beta oscillations are highly present in post central, superior somatosensory gyri, and parieto-temporal gyri and less importantly present in left and right pre-motor and motor areas that can led to speculations of a new "propagation effect" on top of the one described previously regarding the forward motor imagery. On the contrary to the forward propagation effect, this backward effect was more pronounced for the posterior bilateral somatosensory areas than for the anterior pre-motor and motor areas. Given that whole-body motor imagery engages the neural system in a peculiar manner that embrains and embodies task’s constrains^74^, it is possible to speculate that the backward motor imagery, fundamentally relied on the body position in space (i.e. spatial representation) that was constantly updated based on somatosensory representations including vestibular afferences projected into the cerebellum. The participants seemed to utilise their body knowledge (i.e. body representation) to implicitly reconstruct their position and adequately perform backward motor imagery. Coherently with repeated empirical data^75,76^, during the backward imagery movement, more posterior somatosensory activity than anterior motor activity would be required and this because backward anticipatory movement does involve cerebellum activity that provides neural signals associated with somatosensory areas (and does not involve simultaneous visual feedback as the forward motion does). In other words, healthy individuals rely forward motion (real or imagined vs passive or active) to the motor component of the brain, and backward motion (real or imagined vs passive or active) to the somatosensory component of the brain.

Motor and somatosensory components associated with the degree of movement embodiment might differentially influence the multi sensory integration process taking place during the forward and backward mental imagery. In the forward mental imagery condition, beta frontal synchronisation expressing a highly embodied movement would facilitate multi sensory integration process, and beta parietal desynchronisation would attenuate multi sensory integration processes. Such synchronisation/desynchronisation effects are inverted in backward mental imagery condition as this latter expresses a less embodied movement than the former. Accordingly, beta parietal synchronisation would facilitate multi sensory integration process, and beta frontal desynchronisation would attenuate multi sensory integration process. As a whole, the aforementioned neural configuration can be considered as the optimal weighting on multi sensory integration process, which not only involves motor and sensory, including vestibular and visual inputs, but also prior knowledge of whole-body motion, i.e. embodiment*.* Synchronicity between mental motion imagery and embodied experience, which is the case of forward body motion (i.e. common), might assist the multi sensory integration process in frontal areas, whereas asynchronicity between mental motion imagery and embodied experience, which is the case of backward body motion (i.e. unusual), might assist the multi sensory integration process in somatosensory areas. The current results provide some evidence to suggest that whole-body motion on the sagittal-anteroposterior axis would be optimally weighted and processed in multi sensory integration. As such, these findings are not only consistent with recent findings provide evidence for dissociable contributions of different aspects of embodiment to multi sensory integration process^77,78^, but also add new insights to the existing literature by demonstrating that whole-body motion imagery can differentially contribute to the multi sensory integration process depending on the weighting of embodiment.

Considering the imagery of the whole-body motion along the anteroposterior axis, (i.e. both forward and backward motion), it appears from the above that the brain might use precise bilateral beta pre-motor, motor, sensorimotor and somatosensory oscillations. Such observation supports the assumption according to which beta oscillations have a functional and/or conducive role in imagined but also in actual movement. With this in mind, it can be speculated that beta frontal and parietal oscillations may reflect both afferent and efferent copy mechanisms during whole-body motion imagery that might reflect participants’ a priori knowledge of body anteroposterior passive or active motion. Intrinsically, frontal (i.e. pre-motor and motor) and parietal (somatosensory) beta oscillations might depict brain signals associated with the representations of an already performed movement, that is, the whole-body sagittal-anteroposterior motion: the participants were instructed to imaginatively recreate a whole-body passive or active motion experience (including the one they learnt during the mental preparation and training phases of the study). Motor representations of the whole-body would constitute part of the corticocortical network, associated with pre-motor, motor and somatosensory areas involved in the "mental comparison", of the "ordered" (i.e. motion direction indicated by the direction-phrase on the computer screen) with the "memorised" planned and executed whole-body motion.

The conjunction analysis of all recorded brain dynamics associates with the mental imagery of whole-body motion (i.e. forward and backward) revealed beta neural activation in the bilateral pre-motor and motor areas, bilateral primary somatosensory gyrus and bilateral superior and inferior parietal areas. These regions have previously been reported during action observation and are associated with the Action Observation Network (AON)^79^. In the current study, the pre-motor and motor imagery of the whole-body motion embraces several sensorimotor and somatosensory brain areas that are also involved when participants actually perform^80^ or observe the actions performed by others^81^. Taken together, these results might indicate that the AON network would not only integrate the observed actions of others, but also the imagined actions within one’s own personal repertoire. Such speculation seems consistent with the suggestion that brain infers actions (i.e. segmental or global actions) by taking into consideration one’s own motor^82^, somatosensory and somatognosic representations^64,83^. In other words, the mental imagery of whole-body motion and the observation of body actions appear to be pre-motor, motor and somatosensory in essence. When whole-body perception is concerned, studies reported specific activations in extra striate body area, the entire^84^, or mid-fusiform gyrus^85,86^, which is not the case in the current study. The difference between the present study and the above studies resides in the nature and the complexity of the task itself. In the previous studies the participants were instructed to look at motionless bodies that required the analysis and interpretation of visual afferent information of an already located object (i.e. the body and the associated body representation). In the current study, the participants were instructed to imagine moving their body along the sagittal-anteroposterior axis after specific physical and mental training that necessitates the integration of multi sensory information including motor, sensory, and somatosensory information at least.

One potential limitation of the study is due to the nature of the paradigm, which is purely experimental where the participants had to imagine several sequences of forward and backward whole-body motion without executing them apart from during the mental preparation phase. This signifies that the current paradigm did not allow for the investigate of direct comparisons between whole-body representations and whole-body execution. Furthermore, whole-body imagery was restricted to the sagittal-anteroposterior axis. Future studies could include vertical and lateral axes in real, imagined and virtual conditions and consider healthy and neurological populations.

When outlining the current task, it can be concluded that the simulation of the whole-body motion in the imagination consists of a conscious and challenging action that necessitates the activation of the gathered representation of that specific action in each trial. Given the nature of the task, whole-body motion along the anteroposterior axis, developed though substantial perceptual expertise of everyday (passive or active) practice, the mental simulation of which, is associated with the physical execution and therefore the motor, somatosensory and vestibular information is omnipresent and incorporated in the mental representation of the body and its movement. The mental representations individuals build are based on their own experience and expertise and this undeniably influences the way they developed the imagined (whole-body) action in their mind. When they are involved in mental imagery, they re-build the motor action based on their own representations. As per the procedure, in the current study, the participants were trained to do the task before performing it. In that context, the action of simulation during the experimental phase and the gathered (in everyday life and also training phase) motor representation in the imagination share common kinaesthetic components, that is, motor, sensory and somatosensory including vestibular components, that in turn, generate somatosensory and kinesthetic embodied representations. These appear to be mirrored in the neural signatures of the corticocortical frontal and parietal areas reported by the present study.

## Data Availability

The participants did not provide consent for their data to be utilised for different purposes for those described in the original study aims and therefore the datasets cannot be made publicly available. Although de-identified versions of the datasets used for the current study may be made available on reasonable request. Contact person: Irini Giannopulu.
